# Treatment of hypertension with angiotensin‐converting enzyme inhibitors or angiotensin receptor blockers and resting metabolic rate: A cross‐sectional study

**DOI:** 10.1111/jch.14392

**Published:** 2021-11-30

**Authors:** Pablo B. Pedrianes‐Martin, Marcos Martin‐Rincon, David Morales‐Alamo, Ismael Perez‐Suarez, Mario Perez‐Valera, Victor Galvan‐Alvarez, David Curtelin, Pedro de Pablos‐Velasco, Jose A.L. Calbet

**Affiliations:** ^1^ Department of Endocrinology and Nutrition Hospital Universitario de Gran Canaria Doctor Negrín Las Palmas de Gran Canaria Spain; ^2^ Research Institute of Biomedical and Health Sciences (IUIBS) University of Las Palmas de Gran Canaria Las Palmas de Gran Canaria Canary Islands Spain; ^3^ Department of Physical Education University of Las Palmas de Gran Canaria Las Palmas de Gran Canaria Canary Islands Spain; ^4^ Department of Physical Performance Norwegian School of Sport Sciences Oslo Norway

**Keywords:** angiotensin, exercise, obesity, overweight, resting energy expenditure, women

## Abstract

Hypertension in obese and overweight patients is associated with an elevated resting metabolic rate (RMR). The aim of this study was to determine whether RMR is reduced in hypertensive patients treated with angiotensin‐converting enzyme inhibitors (ACEI) and blockers (ARB). The RMR was determined by indirect calorimetry in 174 volunteers; 93 (46.5 %) were hypertensive, of which 16 men and 13 women were treated with ACEI/ARB, while 30 men and 19 women with untreated hypertension served as a control group. Treated and untreated hypertensives had similar age, BMI, physical activity, and cardiorespiratory fitness. The RMR normalized to the lean body mass (LBM) was 15% higher in the untreated than ACEI/ARB‐treated hypertensive women (*p* = .003). After accounting for LBM, whole‐body fat mass, age, the double product (heart rate x systolic blood pressure), and the distance walked per day, the RMR was 2.9% lower in the patients taking ACEI/ARB (*p* = .26, treatment x sex interaction *p* = .005). LBM, age, and the double product explained 78% of the variability in RMR (*R* ^2^ = 0.78, *p* < .001). In contrast, fat mass, the distance walked per day, and total T4 or TSH did not add predictive power to the model. Compared to men, a greater RMR per kg of LBM was observed in untreated hypertensive overweight and obese women, while this sex difference was not observed in patients treated with ACEI or ARBs. In conclusion, our results indicate that elevated RMR per kg of LBM may be normalized by antagonizing the renin‐angiotensin system.

## 1 INTRODUCTION

Resting metabolic rate (RMR) is increased in hypertension^1^, ^2^, ^3^ likely due to increased sympathetic^4^, ^5^ and renin‐angiotensin system (RAS) overactivation.^6^ In overweight and obese hypertensives, the increased RMR is explained by an elevated myocardial oxygen consumption due to an increased resting double product (heart rate x systolic blood pressure), combined with differences in body composition.^1^

Angiotensin‐converting enzyme inhibitors (ACEI) and angiotensin blockers (ARB) are a cornerstone in the treatment of hypertension, mainly when administered to patients with increased RAS activity as hypertensives with overweight or obesity.^7^ Despite recent rodent studies showing that RMR is increased by angiotensin II acting on arcuate nucleus neurones,^8^ it remains unknown whether the RAS system's counteraction is associated with reduced RMR. Moreover, sexual dimorphism in blood pressure regulation and metabolism exists in humans,^9^, ^10^ in part explained by sex differences in the RAS.^11^

Therefore, this study aimed to determine whether resting metabolic rate is reduced in hypertensive patients treated with ACEI/ARB after controlling for the confounding effects of lean mass, fat mass, age, and physical activity. We hypothesized that ACEI/ARB treatment would be associated with lower RMR per kg of lean mass in hypertensive patients treated with ACEI/ARB compared with untreated hypertensives of similar age, BMI, and level of physical activity.

## 2 MATERIALS AND METHODS

### 2.1 Patients

One hundred and seventy‐four participants with overweight or obesity volunteered to participate in a study to reduce body weight with exercise and a low‐calorie diet. As inclusion criteria, men and women had to be 18–70 years old with a body mass index (BMI) ≥ 27, without medical contraindications to exercise and smoking less than six cigarettes per day. Patients with glucose intolerance or type 2 diabetes (if diagnosed within the last five years) were also admitted. More details on inclusion/exclusion criteria can be found in the ISRCTN registry (ISRCTN11049554). Data were collected from June to October 2016. The study was conducted per the Declaration of Helsinki after approval by the Local Ethical Committee (Ref. 140187). All subjects received oral and written information about the purposes, risks, and benefits of the study before providing their written consent. Fifty‐one men and forty‐two women had hypertension, which was untreated in 30 men and 19 women that served as a control group. Twenty‐nine hypertensives, 16 men, and 13 women were treated with ACEI or ARB. In the untreated hypertensive group, four men and two women had type 2 diabetes treated with diet and exercise in three men, while the other man and the two women received metformin. Likewise, one man and one woman from the ACEI/ARB‐treated hypertensives had diabetes treated with metformin. In addition, two men and five women from the untreated hypertensive group were on statins, as well as six men and one woman in the treated hypertensive group.

### 2.2 General procedures

A full description of the general procedures has been previously published.^1^ Subjects reported to the laboratory between 7:00 and 9:30 A.M., following a 12‐hour overnight fast. All subjects were requested not to exercise and to refrain from drinking alcohol and caffeinated drinks during the 48 hours preceding the tests. Upon arrival, their body weight and height were measured to the nearest 0.1 kg and 0.1 cm, respectively (Seca, Hamburg, Germany). The weighing scale was calibrated with certified calibration masses of class M1. After that, their body composition was determined by dual‐energy X‐ray absorptiometry (Lunar iDEXA, General Electric, Wisconsin, USA).^12^ Then, their blood pressure was measured in triplicate (Omron M3 Intellisense HEM‐7131‐E, Hoofddorp, Netherlands) after a 5‐minute seated‐period according to the American Heart Association recommendations.^13^ This was followed by assessing their resting metabolic rate (RMR) by indirect calorimetry, as explained in the next section. Following the RMR test, a 22‐G cannula was inserted in a heated‐hand vein to obtain arterialized blood and basal blood samples. Right after, they ingested 75 g of glucose to carry out a 2‐hour oral glucose tolerance test. Three hours later, they performed an incremental exercise test to exhaustion to determine their VO_2_max.

### 2.3 Resting metabolic rate

Resting metabolic rate (RMR) was measured by indirect calorimetry (Vmax N29; SensorMedics, Yorba Linda, California, USA or Vyntus CPX (Jaeger‐CareFusion, Hoechberg, Germany) at an ambient temperature of 23–26°C.^14^ The metabolic carts were calibrated immediately before each test according to the manufacturer instructions, using certified high‐grade calibration gases. The Vmax N29 SensorMedics has been validated for indirect calorimetry by the ethanol‐burning test. In our laboratory, both metabolic carts overestimated the stochiometric RQ of butane combustion, the Vmax N29 by 2.8% and the Vyntus by 1.5%, with a coefficient of variation below 1% in both cases. All Vyntus CPX data were transformed into Vmax N29 data for further analysis, using values obtained with both analyzers in a parallel cross‐calibration study.

The RMR was assessed for 30 minutes in a well‐ventilated room while the subjects laid supine on a comfortable laboratory stretcher. Participants were instructed to avoid talking and remain motionless. Oxygen uptake (VO_2_) and carbon dioxide production (VCO_2_) were measured breath‐by‐breath for 20 minutes after an initial 10‐minute habituation period using a face mask. For further analysis, the data were averaged every 20 seconds. All 20‐second averages with VO_2_ values deviating from the mean more than two SD were discarded. Then, the mean VO_2_ and VCO_2_ values recorded during a 10‐minute period with steady VO_2_ were averaged to calculate the daily resting energy expenditure.^15^

### 2.4 Maximal oxygen uptake

Subjects performed an incremental exercise test with verification on a cycle ergometer (Corival, Lode, Netherlands). The test started with a load of 20 W increased by 10 W every 3 minutes until the respiratory exchange ratio (RER) was ≥ 1.00. After that, the cycle ergometer was unloaded, and the subjects continued pedaling at low cadence for 2 minutes. Subsequently, the load was increased to the intensity at which an RER of 1.00 was reached and raised by 10 W (women) or 15 W (men) every minute until exhaustion. Then, the cycle ergometer was unloaded while the subjects continued pedaling at a slow speed (40‐50 rpm) to facilitate recovery. At the third minute of the recovery phase, a verification test was started at the intensity reached at exhaustion +5 W during 1 minute, and incremented by 5/4 W in men/women, every 20 seconds, until exhaustion. During the tests, the participants were instructed to maintain pedaling rates close to 70 rpm. Gas exchange data were averaged every 20 seconds, and the highest 20‐second averaged VO_2_ value recorded during the entire test was taken as the VO_2_max.^37^ The VO_2_max results are reported in absolute values (mL/min), normalized to body mass (mL/kg/min), and normalized for RMR (to calculate the corresponding metabolic equivalents or METS).

### 2.5 Physical activity

Participants were equipped with a Garmin Vivofit activity tracker (micro‐electromechanical triaxial accelerometer) (Garmin International Inc., Olathe, KS, USA) to record their physical activity during at least four consecutive days, including two weekend days. Participants' characteristics (gender, age, weight, and height) were introduced into the Garmin Connect website and synchronized with the activity tracker as recommended by the manufacturer. Patients were instructed how to wear the device on the non‐dominant wrist.

### 2.6 Assessment of TSH and T_4_

Five mL of blood were collected into serum vacutainer tubes (1 × 5 mL) with coagulation enhancer and splitting gel (Cat. No. 367955, BD Medical Systems, Franklin Lakes, NJ, USA). After ∼ 30 minutes at room temperature to allow for clotting, blood samples were centrifuged at 2000 g at 4°C for 10 minutes, and the serum supernatants stored at ‐80°C until analyzed. The serum concentrations of thyroid‐stimulating hormone (TSH) and total thyroxine (T_4_) in basal blood samples were measured spectrophotometrically with an enzyme‐linked immunosorbent assay (ELISA) kit (Antibodies‐online, Cat. No. ABIN2773773, Aachen, Germany) in a sub‐sample of forty‐five participants. These participants were randomly assigned from the untreated HTA and the ACEI/ARB groups, maintaining the same proportion from the total number of participants belonging to each group, and conserving the same proportion of men and women within each group. These assays had a sensitivity of 0.078 μIU/mL for TSH and 0.4 μg/dL for T_4_. The measurements were performed according to the manufacturer´s protocol. The intra‐ and inter‐assay coefficients of variation for these assessments are 7.0 % and 7.7 % for TSH and 4.7 % and 5.4 % for T_4_, respectively.

### 2.7 Statistical analysis

The sample size required to show a 10 % between‐groups difference in RMR was estimated to be 26 patients per group, assuming that the standard deviations (SD) of RMR represents approximately 16 % of the measured value^16^ (effect size: 0.8, at α = 0.05 and 1‐β = 0.80; G*Power v.3.1). Results are reported are means ± SD. Firstly, the Kolmogorov‐Smirnov test was run to check for a Gaussian distribution. Data departing from normality were transformed logarithmically before further analysis. Unpaired *t* tests were applied to compare the mean characteristics of the 78 volunteers with hypertension (untreated and under pharmacological treatment with ACEI or ARB) (Table 1). ANOVA was used to determine whether differences in RMR existed between untreated and ACEI/ARB‐treated hypertensives with sex as a between‐individual factor (Table 2 and 3). This analysis was followed by ANCOVA, with FFM, FM, age, double product, and physical activity as covariates. Besides, a multiple regression analysis was performed to determine which variables predict RMR. Values are reported as the mean ± standard deviation (SD) unless otherwise stated. Statistical significance was set at *p* < .05. The statistical analyses were performed using IBM SPSS v.26.0 for Apple Computers (IBM, New York, USA).

**TABLE 1:** Characteristics of the study population

	Men (n = 46)			Women (n = 32)			
	Mean ± SD	Range (min‐max)		Mean ± SD	Range (min‐max)		*p*
Age (years)	43.5 ± 9.7	27.7	67.9	43.0 ± 11.4	19.8	62.5	.82
Weight (kg)	106.6 ± 13.0	83.6	137.3	89.6 ± 12.4	74.1	135.1	<.001
Height (cm)	177 ± 7	161	190	163 ± 6	150	175	<.001
BMI (kg/m^2^)	34.0 ± 2.5	29.6	39.9	33.5 ± 3.1	28.1	45.4	.43
Body fat (%)	37.7 ± 4.7	28.9	51.3	47.6 ± 4.2	39.4	56.1	<.001
Total lean mass (kg)	63.1 ± 8.8	48.1	89.7	44.2 ± 5.0	38.0	56.4	<.001
VO_2_max (mL.min^−1^)	2747 ± 536	1609	3841	1827 ± 382	1249	2757	<.001
VO_2_max (mL.kg^−1^.min^−1^)	25.9 ± 4.5	18.4	34.6	20.5 ± 4.0	14.0	28.2	<.001
VO_2_max (mL.kg LM^−1^.min^−1^)	43.6 ± 6.7	31.2	58.8	41.2 ± 6.4	30.5	55.5	.12
METS (VO_2_max.RMR^−1^)	9.9 ± 1.7	6.4	13.8	8.5 ± 1.4	5.9	12.1	<.001
Distance (km.day^−1^)	7.6 ± 2.9	2.7	15.2	5.7 ± 2.1	2.1	9.3	.004
Steps.d^−1^	11068 ± 3309	5008	20675	10150 ± 2572	5345	14786	.20

BMI, body mass index; kg LM, kg of whole‐body lean mass; Distance, distance walked or run every day; Steps.d^−1^, number of steps walked per day. METS, metabolic equivalents achieved during the incremental exercise to exhaustion; RMR, resting metabolic rate. Analysis based on unpaired t‐test.

**TABLE 2:** Body composition, fitness, and physical activity

		Untreated HTA (M/W: 30/19)	ACEI/ARB(M/W: 16/13)	ANOVA		
				Treat	sex	Sex × Treat
Age (years)	M	41.4 ± 9.4	47.5 ± 9.2	0.069	0.63	0.52
	W	41.8 ± 10.9	44.7 ± 12.3			
Weight (kg)^a^	M	106.0 ± 11.4	108.5 ± 15.9	0.84	0.001	0.72
	W	88.7 ± 9.6^§^	90.4 ± 16.2^§^			
Height (cm)	M	176 ± 7	177 ± 8	0.86	0.001	0.62
	W	164 ± 6^§^	163 ± 6^§^			
BMI (kg.m^−2^) ^a^	M	34 ± 3	34 ± 2	0.85	0.38	0.94
	W	33 ± 2	34 ± 4			
Body fat (%)	M	38.0 ± 5.0	36.9 ± 4.1	0.88	0.001	0.38
	W	47.3 ± 3.6^§^	48.1 ± 4.9^§^			
Total lean mass (kg)^a^	M	62.0 ± 6.4	65.1 ± 12.1	0.80	0.001	0.31
	W	44.5 ± 4.6^§^	43.6 ± 5.7^§^			
VO_2_max (mL/min)	M	2764 ± 454	2717 ± 679	0.33	0.001	0.57
	W	1899 ± 391^§^	1722 ± 359^§^			
VO_2_max (mL.kg^−1^min^−1^)	M	26.3 ± 4.8	24.9 ± 4.0	0.13	0.001	0.87
	W	21.2 ± 3.5^§^	19.5 ± 4.5^§^			
VO_2_max (mL.kg LM^−1^.min^−1^)	M	44.8 ± 7.2	41.5 ± 5.3	0.045	0.001	0.91
	W	42.5 ± 5.9	39.5 ± 6.9			
Distance (km.day^−1^)	M	7.8 ± 2.8	7.1 ± 3.1	0.19	0.007	0.89
	W	6.1 ± 2.2^§^	5.2 ± 2.0			
Steps.d^−1^	M	11 258 ± 3517	10 701 ± 2946	0.24	0.21	0.67
	W	10 648 ± 2423	9459 ± 2707			

M, men, W, women; HTA, hypertension; BMI, body mass index; VO_2_max, maximal oxygen uptake;

* Compared to the untreated hypertensives;

^§^ *p* < .05 women compared to men;

^a^ Statistical analysis after logarithmic transformation. Based on ANOVA (no covariates introduced), Treat: main effect for treatment; Sex: main effect for differences between men and women; Sex × Treat: sex by treatment interaction.

**TABLE 3:** Blood pressure, metabolic variables, end resting energy expenditure

				ANOVA		
		Untreated HTA (M/W: 30/19)	ACEI/ARB (M/W: 16/13)	Treat	sex	Sex × Treat
Systolic BP (mmHg)	M	134 ± 12	140 ± 17	0.75	0.065	0.12
	W	133 ± 10	129 ± 15			
Diastolic BP (mmHg)	M	86 ± 9	87 ± 10	0.72	0.044	0.25
	W	84	±	4	81 ± 9	
MAP (mmHg)	M	102 ± 8	105 ± 11	0.97	0.024	0.12
	W	100	±	5	97 ± 11	
Resting HR (beats.min^−1^)	M	65.3 ± 8.3	68.8 ± 13.3	0.82	0.70	0.062
	W	68.5 ± 6.9	63.9 ± 6.9			
Double Product (Beats.min^−1^.mmHg)	M	8716 ± 1307	9696 ± 2658	0.82	0.27	0.03
	W	9113 ± 1273	8267 ± 1602			
Plasma Glucose (mM) ^a^	M	5.5 ± 0.7	5.6 ± 0.7	0.54	0.038	0.75
	W	5.2 ± 0.6	5.3 ± 0.4			
Plasma Insulin (μU.mL^−1^)^a^	M	9.7 ± 5.0	11.4 ± 4.6	0.40	0.40	0.32
	W	9.8 ± 5.4	9.9 ± 6.6			
HOMAIR^a^	M	2.4 ± 1.4	3.0 ± 1.6	0.37	0.23	0.32
	W	2.3 ± 1.2	2.3 ± 1.6			
Insulinogenic Index ^a^	M	0.8 ± 0.7	0.8 ± 0.6	0.85	0.95	0.39
	W	0.9 ± 0.5	0.9 ± 0.7			
Disposition Index ^a^	M	3.0 ± 2.4	2.1 ± 1.2	0.32	0.34	0.51
	W	3.7 ± 2.5	3.0 ± 2.4			
Matsuda^a^	M	4.2 ± 2.4	3.1 ± 1.3	0.18	0.22	0.61
	W	4.5 ± 2.3	3.9 ± 1.6			
TSH (μUI.mL^−1^)^a^, ^b^	M	1.0 ± 0.8	1.4 ± 0.9	0.11	0.11	0.71
	W	1.0 ± 0.8	1.2 ± 0.7			
Total T4 (μg.dL^−1^)^b^	M	7.3 ± 1.4	7.3 ± 0.9	0.31	0.71	0.27
	W	7.8 ± 1.1	8.6 ± 1.0			
RMR (Kcal.day^−1^)^a^	M	1943 ± 229	2085 ± 449	0.16	0.001	0.006
	W	1647 ± 284^§^	1404 ± 303^§^ ^,^ *			
RMR (Kcal.LM^−1^.day^−1^)	M	31.5 ± 3.1	32.2 ± 4.6	0.026	0.007	0.005
	W	36.9 ± 4.4^§^	32.0 ± 4.0*			

BP, blood pressure; MAP, mean arterial pressure; RMR, resting metabolic rate; M, men, W, women; HTA, hypertension;

* Compared to the untreated hypertensives;

^§^ *p* < .05 women compared to men;

^a^ Statistical analysis after logarithmic transformation. All analysis except TSH and total T4, were based on ANOVA (no covariates introduced), Treat: main effect for treatment; Sex: main effect for differences between men and women; Sex × Treat: sex by treatment interaction.

^b^ Statistical analysis adjusted for age and percentage of body fat.

For TSH and total T4, No. = 17 (9 M/8W) for the untreated HTA group and N = 28 (17 M/11W) for ACEI/ARB group.

## 3 RESULTS

The general characteristics of the patients studied are shown in Tables 1 and 2. Age and BMI were similar in both sexes. Women had a higher percentage of body fat than men. Cardiorespiratory fitness was better in men than women, although the differences were not statistically significant after accounting for lean body mass differences. Men were more physically active than women (Table 1). Twenty‐nine patients, 16 men and 13 women were under treatment with ACEI/ARB (Table 2). The patients treated with ACEI/ARB had a slightly lower VO_2_max normalized to the whole‐body lean mass than the hypertensives without pharmacological treatment (Table 2). Men had marginally higher diastolic and mean arterial pressure values than women. Despite the pharmacological treatment, the resting BP values were similarly elevated in both groups. No significant differences were observed in resting HR or double product (systolic BP x HR) between the two, although there was a significant double product by treatment interaction (Table 3). No significant differences were observed between treated and not treated hypertensives in insulin sensitivity or thyroid function (Table 3).

**FIGURE 1:**
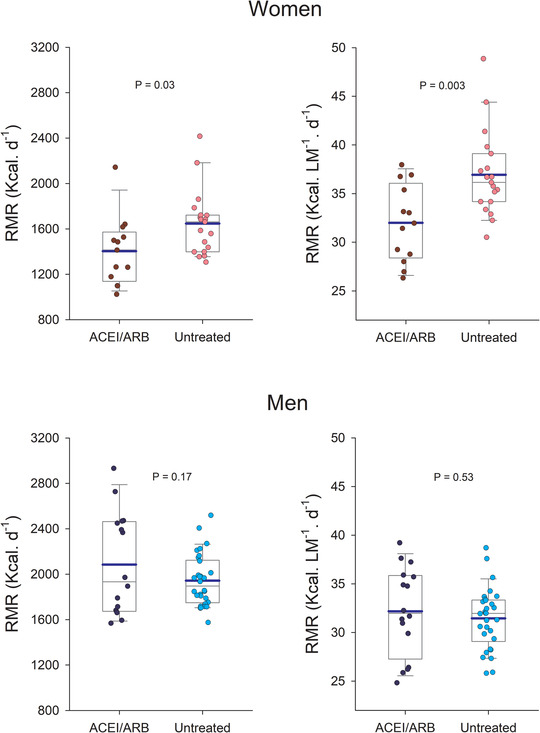
Resting metabolic rate (BMR) in overweight women and men with hypertension treated with angiotensin‐converting enzyme inhibitors (ACEI) and blockers (ARB) or who were untreated. The extremes of the whiskers represent the limits of the 5^th^ and 95^th^ percentiles, respectively; the thin and thick horizontal lines inside the boxes correspond to the mean and median values, respectively; and the lower and upper limits of the box delimit the 1^st^ and 3^rd^ quartiles, respectively. N = 78, 29 were ACEI/ARB treated patients (Men/Women: 16/13) and 49 untreated hypertensives (Men/Women: 30/19). “*p*” values represent the comparison between treated and untreated hypertensives

The RMR in absolute values was larger in men than women (Table 3 and Figure 1). However, when expressed as kcal.d^−1^. kg LM^−1^, the values were marginally higher in women than men due to differences in the untreated group. Consequently, the RMR normalized to the whole‐body lean mass was 15 % higher in the untreated hypertensive women (Table 3 and Figure 1).

After accounting for lean body mass as a covariate, the estimated mean RMR was 6 % lower in the group receiving ACEI/ARB (1718, CI: 1644–1795; and 1828, CI:1766‐1892 kcal.day^−1^, in treated and untreated hypertensives, *p* = .028, Treatment x sex interaction *p* = .004). Adding the whole‐body fat mass as a covariate did not change the picture (1718, CI: 1644–1795; and 1828, CI:1766‐1892 kcal.day^−1^, in treated and untreated hypertensives, *p* = .032, Treatment x sex interaction *p* = .005). After adding age as a covariate, the observed differences in RMR were not statistically significant (1734, CI: 1663–1803; and 1803, CI:1742‐1862 kcal.day^−1^, in treated and untreated hypertensives, *p* = .16, Treatment x sex interaction *p* < .001). Then, we included the double product as a covariate, and no significant differences were observed after accounting for the four covariates (1734, CI: 1667–1807; and 1799, CI:1742‐1862 kcal.day^−1^, in treated and untreated hypertensives, *p* = .15, Treatment x sex interaction *p* = .003). Afterwards we added to the model the distance walked per day, and the observed 2.9 % lower RMR in the patients taking ACEI/ARB was not statistically significant (1738, CI: 1667–1811; and 1795, CI:1734‐1854 kcal.day^−1^, in treated and untreated hypertensives, *p* = .26, Treatment x sex interaction *p* = .005). Finally, we added to the model the total T4 serum concentrations, and the observed 3.8 % lower RMR in the patients taking ACEI/ARB was not statistically significant (1750, CI: 1652–1849; and 1816, CI:1734‐1897 kcal.day^−1^, in treated and untreated hypertensives, *p* = .33, Treatment x sex interaction *p* = .017). After accounting for the six covariates, significant statistical sex x treatment interaction indicated that ACEI/ARB treatment might have attenuated the RMR in women. Similar results were obtained by adding TSH or both T4 and TSH to the model.

Multiple regression analysis showed that lean body mass, age and the double product explain 78 % of the variability in RMR (*R* ^2^ = 0.78, *p* < .001). In contrast, fat mass, the distance walked per day, and total T4 or TSH did not add predictive power to the model (Table 4).

**TABLE 4:** Factors predicting resting metabolic rate (Kcal/ day) in hypertensive patients with or without treatment with ACEI/ARB

Predictor	Estimate	SE	t	*p*	Standardized Estimate (β)	*R* ^2^
Intercept ^a^	1.98748	0.2157	9.22	< .001		
Log Total lean mass (kg)	0.75464	0.1116	6.76	< .001	0.755	0.688
Age (years)	‐0.00213	5.94E‐04	‐3.59	< .001	‐0.233	0.727
Double Product (Beats.min^−1^.mmHg)	0.00000638	0.00000319	2	.049	0.118	0.750
HTA treatment x Sex (interaction)	0.06956	0.0232	3	.004	0.738	0.783
HTA Treatment ^a^	‐0.02069	0.0155	‐1.33	.188	‐0.219	
Sex ^a^	‐0.04135	0.0268	‐1.54	.127	‐0.439	

The resting metabolic rate was logarithmically transformed; N = 78; Sex: Men = 1, Women = 2; hypertension (HTA) treatment with ACEI/ARB = 1, otherwise = 2;

^a^ Represents reference level (Men = 1 and treated with ACEI/ARB = 1).

## 4 DISCUSSION

This study shows that hypertensive overweight or obese women have a greater RMR than men. Treatment of hypertensive overweight and obese patients with ACEI/ARB is associated with a slightly lower RMR. However, the observed differences between treated and untreated patients were reduced to 2.9 % and were not statistically different after accounting for between‐groups differences in total lean mass, fat mass, age, the double product, and physical activity (distance walked per day). Nevertheless, significant sex by treatment interaction remained, indicating that in hypertensive overweight or obese women antagonizing angiotensin II might reduce RMR.

### 4.1 Untreated hypertensive women have a slightly higher RMR than untreated men

Previous studies in healthy humans have reported similar lean body mass normalized RMRs in men and women.^16^, ^17^, ^18^, ^19^ In addition, a small study reported a reduction of lean body mass normalized RMR in hypertensive women after a 10 % weight loss, while it remained unchanged in normotensive women.^20^ In the present cohort, hypertensive women had higher RMR‐normalized to lean body mass than men, which does not seem mediated by the larger fat mass of women than men, since when fat mass was included as a covariate, the differences in RMR persisted. Several factors are implicated in the regulation of RMR, among which the sympathetic nervous system (SNS) and RAS play important roles.^8^, ^21^ The SNS increases brown adipose tissue heat production and facilitates the action of thyroid hormones.^22^ SNS overactivity promotes ATP consumption by stimulating futile cycles and increasing Na^+^‐K^+^ pump and SERCA energy expenditure.^22^ Besides, sympathetic overactivity increases resting heart rate and blood pressure, contributing to elevating RMR by increasing the heart's energy consumption.^1^

One of the mechanisms that could explain a larger RMR per kg of whole‐body lean mass in women is a potentially higher SNS activity in hypertensive obese women.^4^, ^23^, ^24^, ^25^ Besides, greater SNS responsiveness to the cold pressor test measured as increased MSNA has been reported in 60‐year‐old women compared to men of similar age.^26^ Although insulin increases MSNA,^27^, ^28^ basal insulin concentrations were similar in men and women, regardless of ACEI/ARB treatment (Table 3).

Angiotensin II, which is increased in obesity,^29^ has been shown to stimulate sympathetic activity.^30^ In the present investigation, no significant differences in RMR per kg of LM were observed between men and women after treatment with ACEI/ARB, indicating that inhibition of angiotensin II might normalize RMR in hypertensive women. Part of ACEI/ARB's effect is likely due to the reduction of the double product, which has been shown to contribute to increased RMR observed in untreated hypertensives.^1^ In agreement, the heart mass has predictive value for the RMR in women.^31^ Besides, a sex difference in the action ACEI/ARB treatment in RMR is also supported by the significant sex x treatment interaction reported in Table 3. The sex dimorphism in response to the ACEI/ARB treatment may be due to sex differences in the balance between angiotensin II (vasoconstrictive and pro‐inflammatory) and its metabolite angiotensin 1–7 (anti‐inflammatory and vasodilatory). Experiments with mice indicate that obesity increases the hypertensive arm of the RAS (AngII/AT1R) but decreases angiotensin 1–7 and ACE2 in males, while opposite effects were observed in females.^32^ In contrast, our data indicate that women with overweight and hypertension have an elevated RMR, which is normalized by antagonizing angiotensin II action.

In agreement with previous studies,^18^, ^19^ basal thyroid hormone concentrations were not associated with resting metabolic rate in this cohort and did not contribute to explaining the increased resting metabolic rate of untreated hypertensive women in the present investigation.

### 4.2 Antagonizing angiotensin II and RMR

Angiotensin II binds to two G‐protein Coupled Receptors (GPCRs), angiotensin type 1 (AGTR1) and type 2 (AGTR2) receptors. In rodents, two isoforms of the AGTR1 termed AGTR1a and ANGTR1b receptors are expressed.^33^, ^34^ Stimulation of AGTR1a in neurones of the arcuate nucleus by angiotensin II increases both blood pressure and RMR in rodents.^6^, ^35^ Likewise, in rats overexpressing the human renin gene RMR is increased.^36^ In theory, this action of angiotensin II could be mediated through the AGTR1 in humans,^8^ but experimental evidence is lacking. The fact that treatment with ACEI/ARB is associated with normalized RMR in hypertensive women with overweight or obesity supports a thermogenic effect of angiotensin II in humans. In men, ACEI/ARB treatment was not associated with lower RMR may be related to the greater degree of adiposity in women than men (48 vs 38 %), which may facilitate a greater activation of RAS in women. Nevertheless, to definitively establish the role played by adiposity on the effects of ACEI/ARB treatment in RMR, new longitudinal studies will be required in lean and obese hypertensives.

Hypertension is associated with insulin resistance.^2^ However, no association was observed in the present cohort between insulin sensitivity and blood pressure status after accounting for differences between treated and untreated patients in physical activity or cardiorespiratory fitness.^1^

### 4.3 Limitations

The main limitation of this study relies on its cross‐sectional nature and the small sample size. In addition, although the groups were well‐matched by blood pressure, age, BMI, physical activity, and cardiorespiratory fitness, women had a higher percentage of body fat than men. These results will need confirmation with longitudinal studies to establish whether ACEI/ARB treatment may lower more RMR in women than men. In so doing, the information reported in the present investigation may be helpful to estimate the appropriate sample size.

## 5 CONCLUSIONS

In summary, hypertensive overweight or obese women have a greater RMR per kg of lean body than men. This sex difference is not observed in men and women treated with angiotensin‐converting enzyme inhibitors or angiotensin receptor blockers. Our results indicate that elevated RMR per kg of lean body mass may be normalized by antagonizing the RAS.

## CONFLICT OF INTEREST

The authors declare that they have no conflict of interest.

## AUTHOR CONTRIBUTIONS

PPV and JAC conceptualization; PPV, MMR, DMA, and JAC and supervision; PPM, MMR, DMA, IPS, MPV, VGA, DC, PPV, and JAC participated in data collection, analysis, and interpretation of results; PPM, MMR, and JAC wrote the first version of the manuscript; ALL authors contributed comments and approved the final version and are accountable for the for all aspects of the work.
